# Toxidermias

**Published:** 1919-06-07

**Authors:** 


### THE NEW DERMATOLOGY.

### Toxidermias.

Under the term "toxidermia" the vast majority of skin diseases might undoubtedly be classified, but it is preferable to confine the use of it to eruptions produced by substances other than the toxins of bacteria, although this distinction is admittedly artificial. The number of such substances is so large, however, that we can but consider a few of them, and the general principles underlying the pathology of toxic eruptions, with their clinical characteristics as a group, will be discussed, rather than the effects of each individual substance.

The toxidermias offer, perhaps, the most hopeful field for research in the whole realm of dermatology, because in them we can study the effect on the skin of many substances, whose chemical composition and properties can accurately be determined. For this reason, too, they serve as a fitting introduction to the study of eruptions, the causation of which is still a matter of dispute.

According to whether a substance exerts its action after being applied directly to the skin, or after absorption from within, one can distinguish:—

(1) *External toxidermias*, or eruptions directly provoked.

(2) *Internal toxidermias*, or eruptions indirectly provoked.

But, before dealing with the two groups in detail, it is proposed to consider the general pathology of these eruptions.

*The types of eruptions* met with among the toxidermias are very various; in some the lesions are erythematous, in some urticarial, in others purpuric, and in others bullous; again, certain persons under the influence of various toxic substances develop acute generalised eczema, sometimes associated with other types of lesion, sometimes not. These are the commonest cutaneous reactions to toxic substances, and they may be evoked by many things of very different nature. Frequently the reaction is a composite one, different elements being intermingled: an eruption of erythema multiforme, for example, the causes of which are numerous and varied, may consist of erythematous patches, urticarial wheals, bullæ, and even purpuric patches, and it is not uncommon to see a toxic rash partly composed of urticarial elements, and partly of eczematous plaques. These reactions, then, are common, and in no way pathognomonic of the action of any given poison.

On the other hand, an eruption may be specific for certain substances; for example, antipyrine and the bromides sometimes produce lesions which are peculiar to themselves, and arsenical hyperkeratosis may also be considered as specific. And *vice versâ*, as has previously been pointed out, the reaction of a given person may be specific; in other words he may react to all sorts of irritants, both internal and external, by developing a certain type of rash, which is invariably the same; thus the seborrhœic person reacts by developing an eczema, whereas another may always become urticarial. Further, it is often observed that a person will show an idiosyncrasy to the action of one particular substance, to which he will always react in exactly the same way, without there being any necessary correlation between the dose of the irritant and the intensity of the reaction.

When we try to elucidate the pathology of the toxidermias two main considerations present themselves:—(1) The conditions which render certain substances harmful to the skin; (2) the mechanism of their action.

(1) Some substances owe their action to their chemical or physical properties, for example, the caustics, the rubefacients, and those which possess strong oxidising (*e.g.* $H_2O_2$) or reducing (*e.g.* sulphur and chrysarobin) properties. These substances will act in the same way on every skin, though in some instances individual predisposition determines the severity of their action, sulphur, chrysarobin, and "mustard-gas," for example, producing a more intense reaction in seborrhœic subjects than in others. The action of other substances cannot be explained at present by our knowledge of their chemical or physical attributes; with many of these the factor of individual predisposition has to be taken into account.

Of all problems in medicine that of morbid predisposition is one of the most absorbing, and it must be remembered that such predisposition is evident not only towards substances such as we are now considering, but also towards bacterial toxins. As Jadassohn has said, one can apply the term predisposition to two conditions which are up to a certain point distinct: (*a*) *Hypersusceptibility*, whereby a person will show a toxic reaction to a far smaller dose of some substance than is usually required to produce such reaction; (*b*) *Idiosyncrasy*, in which case intolerance is shown to substances which have no toxic action on normal people. As may be imagined, it is often difficult in a given case to judge exactly which of these phenomena is concerned.

Although we are far from really understanding upon what the state of predisposition depends, certain factors which evidently play a part may be indicated. In many cases, as has already been described, anaphylactic sensibility will explain a predisposition, the eruptions produced by the injection of foreign sera, and probably in some instances those following the ingestion of certain foods, and exposure to poisonous plants being examples of this kind. Strictly speaking anaphylactic susceptibility must be acquired, but some cases of predisposition are congenital and even familial.

It seems likely that predisposition is accentuated by certain physiological changes, such as menstruation, pregnancy, the menopause, and puberty; it is usual, for example, for women with rosacea, which is certainly a toxidermia if the word be used in its widest sense, to be worse before and during their menstrual periods, and the so-called herpes

**The New Dermatology**—(*continued*).

gestationis is but dermatitis herpetiformis, recurring in some unfortunate women with each pregnancy.

In other cases a toxidermia would seem in part to be determined by an abnormality of the skin itself, or by diseases of some internal organs. Examples of the former are seen in the liability to " application dermatitis " shown by ichthyotics, that to eczematisation by seborrhœic persons, and the susceptibility of those with acne vulgaris and seborrhœa to the artificial forms of acne, following the ingestion of bromides and iodides, or the application to their skin of substances such as tar, camphorated oil, etc.

And with regard to the influence of diseases of internal organs, it is a well-known fact that people with digestive disorders are very liable to develop intolerance towards certain drugs—*e.g.* bromides and iodides, and those with diseases of the liver and kidneys exhibit the same tendency. It can, indeed, be readily understood that hepatic and renal insufficiency increase the likelihood of toxæmic manifestations, since the liver is the greatest detoxicating organ in the body, and the non-toxicity of many substances depends on the rapidity with which they are excreted in the urine. Hence the importance of testing the renal functions when such drugs as mercury and the organic arsenical compounds are administered.

These, then, are some of the factors which must be considered in investigating a case of toxidermia.

(2) *The mechanism of the action of toxic substances.* It has already been said that there are certain cutaneous reactions which may be evoked by a number of different substances, namely, erythema, urticaria, purpura, bullæ, and eczema—whereas a few only are in any way specific. The common reactions form a natural series, particularly the first-named four, according to their intensity. Thus, erythema is the simplest type of reaction, and in its purest form consists merely of dilation of the vessels of the papillary bodies; if the congestion becomes intense, exudation of plasma occurs, and we get an *urticarial* element added; a further stage is marked by the diapedesis of white corpuscles (papular erythema); still further both red and white corpuscles escape into the tissues, giving rise to *purpuric* spots; and lastly, *bullæ* may be formed in two ways, (*a*) by a local œdema in the dermis under high pressure, but serous fluid either filtering through the malpighian layers and coming to lie under the horny layer (superficial type), or lifting the epidermis entire (deep type); (*b*) by a process known as acantholysis, by which is meant the separation and disintegration of the prickle cells under the pressure of the intercellular exudate. We thus see that these types of lesions represent different degrees of intensity of reaction, and in some toxidermias, as has been said, all types may co-exist at the same time.

It seems probable that these cutaneous reactions are practically always inflammatory in nature, *i.e.*, produced in response to an irritant, acting either externally on the skin, or after absorption from elsewhere in the body, and the type of lesion evoked would seem to depend on the toxicity of the irritant in question, and on the susceptibility of the person exposed. The part played by the vaso-motor system in the production of some erythemata and urticarial eruptions is evident enough, but its action has undoubtedly been exaggerated, and it is to the direct action of toxins on the tissues themselves, probably of a physico-chemical nature, that most of the cutaneous reactions are due.

One point of interest is that different portions of skin show different degrees of susceptibility to a given irritant, for this may provoke, say, an erythema on the face and arms, urticaria of the trunk, and purpura on the legs in the same person.

This probably depends on differences in the chemical composition of the skin structures in different parts of the body; thus Unna has shown that chrysarobin exerts its action most profoundly where the sebaceous glands are numerous, whereas areas in which these are scanty are relatively resistant to its action.

Lastly, it must be remembered that the primary toxic eruption may be considerably modified by the secondary action of micro-organisms present in the skin, particularly those of the pyogenic group, the primary eruption having so altered the skin as to diminish its natural resistance to the active growth of these organisms.

(*To be continued.*)

